# Tincturæ Nucis Vomicæ an Antiperiodic

**Published:** 1875-01

**Authors:** W. W. Fierce

**Affiliations:** Wilkesville, Ohio


### TINCTURÆ NUCIS VOMICÆ AS AN ANTIPERIODIC.

BY W. W. FIERCE, M.D., OF WILKESVILLE, OHIO.

[*Read before the Meigs County (Ohio) Medical Society.*]

Was called, June 20th, by Mr. H. to see his daughter, age ten. On account of press of business, could not reach the patient until the morning of the 21st. The mother of the little girl stated that on the morning of the 18th her daughter complained of feeling unwell, refusing to take any breakfast, and in a short time was attacked with a very severe chill, which lasted two hours or more, and was followed by high fever and delirium. When I reached my little patient, she was just passing through the febrile condition after the fourth chill. I prepared—℞. Tincturæ nucis vomicæ, tincturæ gelseminum, aa. ʒ ss.; aquæ, ℥ ii. Misce. Signa. One teaspoonful every three hours until my return.

23d. Received word from Mr. H. that his little girl was so much better that he did not consider it necessary for me to visit her again, but would report if the patient needed anything more. The chills did not return, consequently there was no further need of medicine.

CASE II.—June 21st, 2 o'clock P.M., visited Mr. S., age twenty-nine, laborer; lives three miles from the above-named patient. Found patient with high fever, muttering delirium, tongue covered with a dirty-brown coat, bowels constipated. Was attacked on the 19th, while at work, with a severe chill, followed in a short time by intense fever, which lasted all night. Thinking the indications good for calomel, I accordingly administered grs. xv., to be followed in five hours with magnesia sulphas q. s. And for the chills and accompanying delirium I gave—℞. Tincturæ nucis vomicæ, tincturæ gelseminum, aa. ʒj.; aquæ, ℥ ii. Misce. Signa. One teaspoonful every eight hours, to be continued until my return.

22d. Patient rested well all night; bowels moved off nicely. Continue the preparation I gave yesterday until it is all taken. Patient did as directed, and there was no return of the chills.

CASE III.—June 30th, Mrs. McL., age fifty-two, living in the same neighborhood of the two just mentioned; has had two chills, followed with fever; no delirium. Patient says she has taken "lots of quinine" at various different times for chills; thinks she would like some other remedy for the ague if I had anything of the kind with me. I prepared—℞. Tincturæ nucis vomicæ, ʒj.; aquæ, ℥ii. Misce. Signa. One teaspoonful every eight hours, to be notified if there is a return of the ague.

July 2d. Slight fever to-day, but no chill; some of the toxical effects of nux vomica quite perceptible. To discontinue the drug until these peculiar symptoms subside, then take the rest of the two-ounce mixture. Saw patient four weeks afterwards, and there had been no return of the ague, and on inquiry I found there had been no return of the symptoms peculiar to strychnia poisoning.

Case IV.—July 14th, was called to see Mr. M., age forty-seven, laborer; found my patient suffering with what he termed "shakes." Contracted the ague in the swamps of Indiana some sixteen months prior to my being called. The general appearance of my patient was unfavorable—tongue covered with a dirty-brown covering; bowels constipated; urine scanty and high-colored; patient considerably emaciated; the skin of the whole body presents very much the appearance of yellow clay; found that he knew what quinine was; states that "I have been taking quinine as a regular diet for fifteen months." The indications were good for calomel; I accordingly administered a full dose, to be followed in a few hours with magnesia sulph. q. s. I then prepared—℞. Tincturæ nucis vomicæ, ʒj.; aqua, ℥ii. Misce. Signa. One teaspoonful every three hours, with the understanding that if certain symptoms (which I named) supervene, to discontinue the preparation and report to me; but nothing of the kind occurred. The ague was arrested for two weeks, when it returned in a milder form. I then repeated the nux mixture, since which time Mr. M. has had no more ague.

Case V.—Mr. D. called at my office and says his daughter, age eighteen, is having the "shakes" every other day, and heavy fever follows each "shake." Not having any nux vomica in the office, I sent quinia sulph. grs. xx. Divide in pulveries iv. Signa. One powder every four hours, to report if anything more is needed.

25th. A brother of Miss D. calls and says his sister is shaking again. I sent—℞. Tincturæ nucis vomicæ, ʒj.; aquæ, ℥ii. Misce. Signa. One teaspoonful every three hours, to take all of the two-ounce mixture, unless some unfavorable symptoms should occur.

The ague was arrested promptly, without any of the ill-effects of the nux; neither was there any return of the ague.

CASE VI.—August 18th, was called by Mrs. H., age thirty-five; has had two hard chills, followed with high fever. I prepared—℞. Tr. nux vom., ʒj.; aquæ, ℥ii. Misce. Signa. One teaspoonful every eight hours. The chills were arrested, and the lady has not had any more ague since.

CASE VII.—August 29th, Mr. McJ. calls at the office; his little son, aged five, has had three chills, followed with fever. I sent—℞. Tincturæ nucis vomicæ, ʒss.; aquæ, ℥ii. Misce. Signa. One-half teaspoonful every three hours. The chills were arrested, and there has been no return since.

CASE VIII.—August 30th, was called to see Mrs. F., aged twenty-four; found a well-marked case of ague, and as the tongue looked as if the system needed calomel, I administered a full dose, with instructions to give magnesia sulph. q. s. in five hours. I then prepared—℞. Tincturæ nucis vomica, ʒj.; aquæ, ℥ii. Misce. Signa. One teaspoonful every eight hours. The chills did not return again.

CASE IX.—September 2d, Mr. McL., age twenty-two, laborer; has had two chills, followed with fever. I gave—℞. Tr. nux vom., ʒj.; aquæ distillata, ℥ii. Misce. Signa. One teaspoonful every three hours. The chills returned in one week, caused, as I afterwards learned, by carelessness. Sent the same preparation, since which time there has been no return of the ague.

I might thus go on and enumerate *many more* cases in which I have used tincturæ nucis vomicæ as an antiperiodic, but for the present I consider this unnecessary, for I am well aware of the fact that if any one advances a new idea, or even reëstablishes an old theory, in the science of medicine or surgery, it matters not how tenable that theory may be, such a one is almost always thoroughly hashed up by the fraternity generally; this is a fact that I am well aware of, consequently it is with reluctance that I undertake to publish to the medical world—after three years' experimenting with this drug—that I say it can, without a doubt, be classed as a safe and reliable *antiperiodic.*

My attention was first drawn to this drug as an antiperiodic in the fall of 1871, in Bloomfield, Iowa. My partner (E. J. Shelton,

M.D.) and myself prescribed for our typhoid patients tincturæ nucis vomicæ et acitum phosphoricum dilutum. The patients who took this preparation—and we administered it to some thirty-five or forty cases—made a good recovery without any quinia sulph. This, I say, is what called my attention to the tinct. of nux, and led to my experiments, and from that time to the present I have used it with as good results as I ever obtained from quinia sulph. The question may be asked, Will tincturæ nucis vomicæ fill the place of quinine; or, in other words, will it be applicable in all cases in which that time-honored drug has been used? This question I can answer without hesitation, and I found my answer an actual demonstration, for I have administered it for as many ills (and they are legion) as I ever did quinine, and I must say with better success.

Pereira names a number of diseases in which nucis vomicæ has been used with benefit, and then closed his remarks on the uses of this drug by saying: "The preceding are the diseases in which nux vomica has proved most successful. It has, however, been used in several others (as *intermittent fevers, intestinal worms,* etc.) with occasional benefit." As for the physiological action or "modus operandi" by which nux vomica eliminates from the system the poison which produces intermittent fevers, I will not in this article (as space will not admit) attempt a scientific explanation. Pereira says of nux: "In febrile states of the system, its use is contra-indicated." According to my own experience, nux can be used with perfect safety, even when the system is in a high febrile condition. When administering nucis vomica for ague, I invariably give it during the febrile stage, and it appears to act as a nerve-sedative at such times, instead of being contra-indicated. My opinion is that if it acts in a deleterious manner under such circumstances, the bad effect can be attributed to an idiosyncrasy of the patient."

Such is a brief record of some of my experience and observations with tincturæ nucis vomicæ. I present to the Fellows of the Society these observations, asking you to give them just what merit they deserve and no more; but before casting into oblivion by your criticisms this article, I beg of you to give nux vomica a thorough trial.

